# Banana21: From Gene Discovery to Deregulated Golden Bananas

**DOI:** 10.3389/fpls.2018.00558

**Published:** 2018-04-26

**Authors:** Jean-Yves Paul, Robert Harding, Wilberforce Tushemereirwe, James Dale

**Affiliations:** ^1^Centre for Tropical Crops and Biocommodities, Queensland University of Technology, Brisbane, QLD, Australia; ^2^National Agricultural Research Organisation, Kampala, Uganda

**Keywords:** East African highland banana, staple crop, Uganda, micronutrient deficiency, vitamin A deficiency, pro-vitamin A, carotenoids, biofortification

## Abstract

Uganda is a tropical country with a population in excess of 30 million, >80% of whom live in rural areas. Bananas (*Musa* spp.) are the staple food of Uganda with the East African Highland banana, a cooking banana, the primary starch source. Unfortunately, these bananas are low in pro-vitamin A (PVA) and iron and, as a result, banana-based diets are low in these micronutrients which results in very high levels of inadequate nutrition. This inadequate nutrition manifests as high levels of vitamin A deficiency, iron deficiency anemia, and stunting in children. A project known as Banana21 commenced in 2005 to alleviate micronutrient deficiencies in Uganda and surrounding countries through the generation of farmer- and consumer-acceptable edible bananas with significantly increased fruit levels of PVA and iron. A genetic modification approach was adopted since bananas are recalcitrant to conventional breeding. In this review, we focus on the PVA-biofortification component of the Banana21 project and describe the proof-of-concept studies conducted in Australia, the transfer of the technology to our Ugandan collaborators, and the successful implementation of the strategy into the field in Uganda. The many challenges encountered and the potential future obstacles to the practical exploitation of PVA-enhanced bananas in Uganda are discussed.

## Introduction

Vitamin A (VA) or retinol is an important nutrient which supports vital physiological and developmental functions. Since it cannot be synthesized *de novo*, VA has to be acquired through a diversified diet (Fraser and Bramley, 2004; Kimura et al., 2007; Beyer, 2010; Fitzpatrick et al., 2012). Whereas retinol is derived directly from animal sources, plant-derived pro-vitamin A carotenoids (PVACs) such as α- and β-carotene must first be converted into retinol by the body (van den Berg et al., 2000). The majority of populations living in developing countries depend on starchy food staples such as cassava (*Manihot esculenta*), maize (*Zea mays*), potato (*Solanum tuberosum*), rice (*Oryza* spp.), plantain, and banana (*Musa* spp.) which unfortunately are largely deficient in essential micronutrients such as PVACs.

Vitamin A deficiency (VAD) causes a number of VAD disorders including night and total blindness, premature death (Sommer and Vyas, 2012), and reduced immunity leading to increased risk of childhood infections and high infant mortality (Herbers, 2003; Bai et al., 2011). VAD affects an estimated 190 million pre-school children worldwide, most of whom live in developing countries, with a reported 5.17 million registered cases of clinical or severe levels of VAD (WHO, 2009). It is also estimated that 250,000–500,000 children become blind due to VAD each year, half of whom die within 12 months of losing their sight (WHO, 2009; Barber et al., 2012). In Uganda, 20% of children aged 6 months to 5 years and 19% of women aged 15–49 years suffered from VAD in 2006, predominantly in the low-income demographics within central Ugandan communities, which heavily rely on banana as a staple food (UDHS, 2006).

In the Great Lakes region of East Africa highland bananas (EAHBs) are an important food security crop and the main food staple (Adeniji et al., 2010). In Uganda, it is estimated that 75% of farmers grow bananas contributing to about 7% of global banana and plantain production (Ssebuliba et al., 2006). In addition, Uganda is the largest banana consumer in the world with an estimated per capita consumption of 220–250 kg/year (Tushemereirwe et al., 2006). In rural populations of Uganda where EAHBs form a major and sometime unique part of the diet, 20 μg/g dry weight (dw) β-carotene equivalent (β-CE) is the minimum amount of PVA required in banana fruit to provide 50% of the estimated average requirement (EAR) of VA (Paul et al., 2017). Despite their considerable biodiversity, most EAHB varieties grown in East Africa have low levels of essential micronutrients such as iron (Fe), zinc (Zn), and PVACs (Davey et al., 2009; Fungo et al., 2010). Overreliance on banana in this region, and in Uganda in particular, has contributed to the exacerbation of micronutrient deficiency-related illnesses.

Strategies such as diet diversification, supplementation, and food fortification have been used to help alleviate some of these ailments with varying levels of success (WHO, 2009; Gómez-Galera et al., 2010; Fitzpatrick et al., 2012; Bruins and Kraemer, 2013). However, whereas these interventions are very successful in an urban context where the target population is in the vicinity of service providers, they fail to reach rural communities that are most in need (Dalmiya and Palmer, 2007; Victora et al., 2008; WHO, 2009). As such, in Uganda, VADs in children are relatively low in urban areas but remain elevated in rural communities (UDHS, 2006).

More recently, biofortification has emerged as a complementary, cost-effective, and sustainable approach to deliver micronutrient-dense crops to the poorest and hardest-to-reach communities. This can be achieved through either conventional breeding, where the necessary traits are available within the accessible “breeder’s gene pool,” or through genetic modification. Examples of successful conventional breeding approaches include the biofortification of maize (Egesel et al., 2003; Harjes et al., 2008), sweet potato (Mwanga et al., 2009), and cassava (Ceballos et al., 2012). These biofortified products are already being disseminated in various parts of the world including Uganda (Anderson et al., 2007; Hotz et al., 2012; Talsma et al., 2016). Arguably the most successful example of biofortification through genetic engineering is the development of Golden Rice (GR) (Ye et al., 2000; Paine et al., 2005). Considering the popularity of EAHBs in East Africa, biofortification of this food staple with enhanced levels of PVACs (or other micronutrients) is now believed to be the best long-term, sustainable, and cost-effective strategy to ease the burden of VAD in high risk populations of Uganda. The use of conventional breeding to develop PVA-biofortified EAHBs is constrained by their low fertility and the lack of high PVA EAHB varieties in the known gene pool. Further, any new varieties developed are unlikely to possess the attributes of locally preferred landraces of EAHBs. Genetic modification is therefore the fastest and most reliable approach to improve the existing preferred varieties.

Here, we review the foundations, goals, achievements, future prospects, and challenges of Banana21^1^, a project that undertook the challenges of alleviating micronutrient deficiencies in Uganda by enhancing the nutritional content of its staple food, EAHBs, through genetic modification.

## The Promise of Banana21 – More Nutritious Bananas Made in Uganda, by Ugandan Scientists, for the Ugandan People

Banana21 is one of four original projects funded by the Grand Challenges in Global Health (GCGH) program of the Bill and Melinda Gates Foundation (BMGF) to “Create a Full Range of Optimal, Bioavailable Nutrients in a Single Staple Plant Species.” Banana21 is a collaborative research project between the Centre for Tropical Crops and Biocommodities at Queensland University of Technology (QUT) in Australia and the National Banana Research Program of the National Agricultural Research Organisation (NARO) of Uganda. The project aims to help “Alleviate VAD and iron deficiency anemia through the micronutrient enhancement of the staple food of Uganda, bananas.”

From the outset, it was recognized that the success of the project depended on its objectives being widely understood and accepted in Uganda. As such, we considered it extremely important to train and empower young Ugandan scientists to deliver the project milestones thus ensuring the biofortified genetically modified (GM) bananas were generated in Uganda, by Ugandan scientists, from Ugandan varieties, and for the benefit of the Ugandan people. As a consequence, in addition to scientific discoveries and their applications, Banana21 has been a capacity building project where technology transfer continues to be at the core of every phase, milestone, and decision-making activity.

**FIGURE 1:**
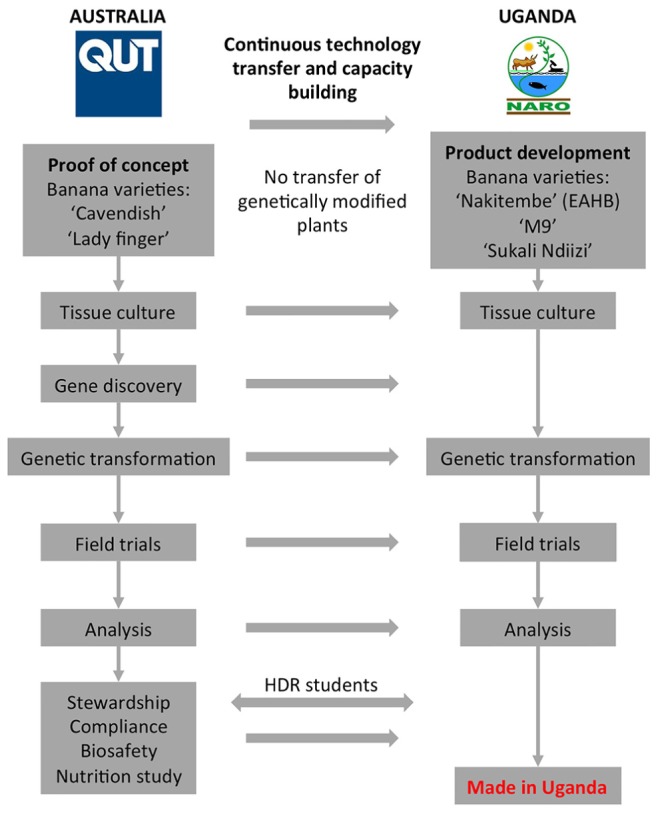
The Banana21 project. EAHB, East African highland banana, HDR, higher degree research.

An overview of the Banana21 project strategy is shown in **Figure 1**. Initially, the proof-of-concept research was developed at QUT using the locally grown dessert banana cultivars “Cavendish.” The QUT components included gene and promoter discovery, tissue culture and transformation, field trials of GM bananas, and downstream fruit sampling and analysis. The development and implementation of a comprehensive stewardship plan was also a key project component. Following proof-of-concept in Australia, the technology and know-how was to be continuously transferred to NARO in Uganda to generate PVA-biofortified local EAHB varieties. Further, it was necessary for the infrastructure to be implemented at NARO to ensure that the GM plants produced would be tested in the laboratory and the field with the consistency and rigor necessary to generate the data required for a GM product deregulation dossier.

## The Challenges and Achievements of Banana21 – Transgenic Biofortified EAHBs With Significantly Higher Fruit PVA Content

### Phase 1 – Early Discovery and Technology Transfer

At the commencement of the project in 2005, the only practical demonstration of PVA biofortification in a staple food crop was GR. The initial GR strategy involved the re-engineering of a carotenoid biosynthesis pathway in normally carotenoid-free rice endosperm by the endosperm-specific [glutelin 1 (gt1) promoter] expression of a daffodil-derived phytoene synthase (*psy*) transgene in combination with the constitutive (CaMV 35S) expression of a bacterial phytoene desaturase (*crtI*) gene. Although successful, the level of carotenoid accumulation in rice endosperm was still considered to be too low for practical exploitation (Ye et al., 2000). Subsequent research revealed that the origin of the *psy* transgene and choice of promoter were important factors affecting PVAC accumulation levels (Paine et al., 2005). This led to the development of Golden Rice 2 (GR2) whereby the use of a maize-derived *psy* transgene and bacterial *crtI*, both under the control of the gt1 promoter, resulted in high levels of PVAC accumulation (Paine et al., 2005). Based on this success, the GR2 strategy was deemed to be the most logical approach to develop VA-biofortified bananas. One of the major initial challenges was the identification of suitable transgenes and promoters for expression in banana. As such, the early phase of the project focused on designing and testing large numbers of expression constructs containing a suite of different promoters and transgene combinations.

Due to the paucity of information regarding transgene expression in banana fruit, it was necessary to assess a range of different promoters for their spatiotemporal activity in banana, particularly fruit. As such, several constitutive promoters were isolated from different sources in addition to promoters that controlled the expression of genes involved in banana fruit development. These were fused to the β-glucuronidase reporter gene (*uidA*), transformed into banana embryogenic cell suspensions (ECS) (Khanna et al., 2004) and transgenic plantlets regenerated. A list of all promoters tested, their origin, and specificity is presented in **Table 1**.

**Table 1:** List of promoters and genes tested in AFT-1 of the Banana21 project.

Promoter abbreviation	Full name	GenBank	Source	Specificity
Ubi	Polyubiquitin	S94466	*Zea mays*	Constitutive
TaBV	Taro bacilliform virus	NC_004450	Taro bacilliform virus	Constitutive
35S	CaMV 35S	AF234316	Cauliflower mosaic virus	Constitutive
Nos	Nopaline synthase	X01077	*Agrobacterium tumefaciens*	Constitutive
Exp1	Expansin 1	AF539640	*Musa acuminata* cv. Cavendish	Fruit
Aco	1-Aminocyclopropane 1-carboxylate oxidase	AF221107	*Musa acuminata* cv. Cavendish	Fruit
Acs	1-Aminocyclopropane 1-carboxylate synthase	AF119096	*Musa acuminata* cv. Cavendish	Fruit
BT-1	Banana bunchy top virus DNA-1	NC_003479	Banana bunchy top virus	Vascular-associated cells
BT-4	Banana bunchy top virus DNA-4	NC_003474	Banana bunchy top virus	Vascular-associated cells
BT-5	Banana bunchy top virus DNA-5	NC_003477	Banana bunchy top virus	Vascular-associated cells
BT-6	Banana bunchy top virus DNA-6	NC_003476	Banana bunchy top virus	Vascular-associated cells
MtPsy2a	Phytoene synthase 2a	Not available	*Musa troglodytarum* cv. Asupina	Unknown

**Gene abbreviation**	**Protein**	**GenBank**	**Source**	**Function**

*MtPsy2a*	Phytoene synthase 2a	JN129487.1	*Musa troglodytarum* cv. Asupina	Carotenoid biosynthesis
*ZmPsy1B73*	Phytoene synthase 1	AY773475.1	*Zea mays* cv. B73	Carotenoid biosynthesis
*PaCrtI*	Carotene desaturase	D90087	*Pantoea ananatis*	Carotenoid biosynthesis
*uidA*	β-Glucuronidase (GUS)	S69414.1	*Escherichia coli*	Reporter gene


In combination/parallel with the promoter characterization study, a range of different transgenes were also assessed. Initially, the strategy used to develop GR2 was adapted to banana and the first generation of expression constructs was made to express the maize phytoene synthase 1 (*ZmPsy1B73*) transgene alone or in combination with the bacterial (*Pantoea ananatis*) carotene desaturase transgene (*PaCrtI*) and controlled by various promoters (**Table 1**). Our efforts also focused on isolating and using PVA-associated cisgenes from banana in the hope of minimizing gene silencing and thus achieving more stable gene expression over several generation. The use of banana-derived cisgenes/s was also considered to be more advantageous from a future deregulation perspective. The biodiversity of banana and plantain is huge especially in Southeast Asia and Papua New Guinea where bananas are believed to have originated (Perrier et al., 2011). As such, the ability of various cultivar to accumulate PVACs in their fruit is also very diverse (Davey et al., 2009; Ekesa et al., 2015). Of particularly interest to us was a small group of bananas originating from the Pacific called the Fe’i bananas (*Musa troglodytarum*) which accumulate extremely high levels of fruit PVACs (Englberger et al., 2003). One such Fe’i banana variety, “Asupina,” became our model cultivar for not only understanding carotenoid metabolism and accumulation in banana fruit but also as the source of a cisgene encoding a phytoene synthase, *MtPsy2a* (Mlalazi et al., 2012; Buah et al., 2016). Following the cloning and molecular characterization of *MtPsy2a*, expression cassettes containing this gene with and without *PaCrtI* and controlled by various promoters were also constructed and used to generate transgenic “Cavendish” banana lines in order to assess the levels of PVA accumulation in fruit.

During Phase 1, an important training program was also initiated whereby several students from Uganda and Kenya commenced their higher degree research (HDR) doctoral studies at QUT. The research projects developed for these students focused primarily around banana tissue culture and transformation technologies so that the knowledge and experience gained in the QUT laboratories would be transferred back to Africa for the benefit of the project. Simultaneously, training of technical staff and capacity building around infrastructure and laboratory equipment began at NARO, Uganda. A major component of this training program was the development of protocols and standard operating procedure (SOP) for generating and transforming ECS of local banana varieties. From the beginning, the Ugandan component of the project focused on establishing ECSs from three popular Ugandan banana cultivars, namely “Nakitembe,” “M9,” and “Sukali Ndiizi” since these were considered the most appropriate target cultivars for biofortification. True EAHB varieties such as “Nakitembe” are the preferred cultural choice among Ugandans and are usually grown in the highlands where disease pressure is minimal. The disease-resistant EAHB hybrid “M9” is less popular but was chosen as it is more productive in the lowlands where diseases such as black Sigatoka are a serious limitation to banana production. “Sukali Ndiizi” was chosen because it is a small sweet banana which is popular among children, the most vulnerable target population.

### Phase 2 – Proof of Concept, Field Trials in Australia and Uganda

The banana fruit PVAC target levels necessary to deliver 50% of the EAR of VA in vulnerable populations was estimated at 20 μg/g dw β-CE. The PVA-biofortification proof-of-concept research was done in Australia using transgenic “Cavendish” and “Lady Finger” bananas as the models with the aim of obtaining the target fruit PVA levels with no changes in agronomical characteristics. One of the major limitations of the project was the lengthy timeframe from transformation of banana ECS through to fruit harvest which is approximately 30 months. This limitation precluded the serial testing of our expression constructs. Consequently, our strategy was to transform “Cavendish” and “Lady Finger” ECS in Australia with a large number of different expression constructs, regenerate transformants, and field trial all the plants in parallel. At the end of Phase 1, between 10 and 30 independents transgenic “Cavendish” banana lines for each of the expression constructs had been generated. Considering the very large number of lines to be tested, and the fact that bananas are a very large crop, it was not practical to conduct a trial in the glasshouse. With these limitations in mind, we assessed these lines directly in the field without prior glasshouse characterization. The first Australian field trial (AFT-1) of GM “Cavendish” banana lines was subsequently established in 2009. This trial contained all available independent lines for each construct but only a single plant per line. A total of 28 constructs were tested, 14 to test promoter activity (as promoter/*uidA* reporter gene fusions) and 14 to test the same promoters in combinations with three transgenes (*MtPsy2a*, *ZmPsy1B73*, and *PaCrtI*) encoding proteins involved in the biosynthesis of PVACs (**Table 1**). This was the first GM banana field trial in Australia and was conducted under a license issued by the Australian Office of the Gene Technology Regulator (OGTR)^2^.

**FIGURE 2:**
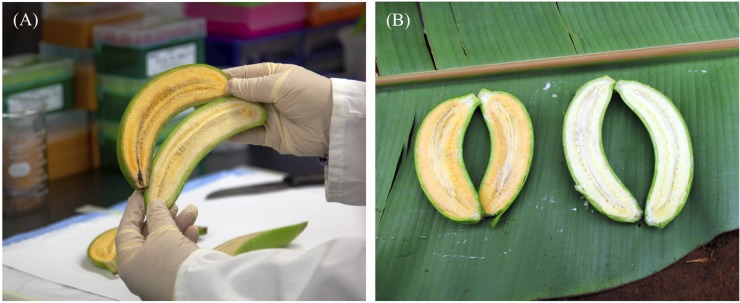
**(A)** PVA-biofortified “Cavendish” banana in Australia and **(B)** PVA-biofortified (left) and wild-type control (right) “Nakitembe” EAHB in Uganda.

Of the 14 promoter/*uidA* fusion combinations tested in the field, three promoters (Ubi, Exp1, and Aco) consistently conferred the strongest levels of GUS expression. The constitutive maize polyubiquitin (Ubi) promoter showed consistently strong activity from the earliest stages of fruit development through to maturity. In contrast, the banana fruit-specific expansin 1 (Exp1) and ACC oxidase (Aco) promoters were only active in the later stages of fruit development (Paul et al., 2017). When we subsequently investigated the accumulation of PVACs during fruit development, a similar trend was seen whereby constitutive expression of either *MtPsy2a* or *ZmPsy1B73* using the Ubi promoter increased PVA accumulation from the earliest stages of fruit development while PVAC accumulation was restricted to the late stages of fruit development when the same transgenes were tested under the control of either the Exp1 or Aco promoters. Analysis of fruit samples from plants transformed with *MtPsy2a*, *ZmPsy1B73*, and/or *PaCrtI* revealed that constitutive (Ubi) expression of *MtPsy2a* resulted in the highest fruit β-CE levels which reached almost 19 μg/g dw in the plant crop (**Figure 2A**). Although this was just below the target level, it nonetheless represented a 11-fold increase over wild-type banana PVA baseline levels and was highly encouraging (Paul et al., 2017).

A second Australian field trial (AFT-2) was planted in September 2012 to test the efficacy of new PVA biofortification constructs as well as to monitor transgene stability in multiple plants of selected lines from AFT-1 through three successive generations. The additional PVA genes and promoters tested in this second field trial (**Table 2**) were tested in combination with the *MtPsy2a* gene under the control of various promoters. Unfortunately, none of the new combinations tested were found to elevate fruit PVA levels as high as those achieved by the constitutive expression of *MtPsy2a* alone in AFT-1. However, an extremely interesting and important outcome from AFT-2 was the observation that fruit PVA levels increased over successive generations in some lines to levels exceeding the target. For example, the fruit PVA levels of a line containing Ubi-*MtPsy2a* was found to increase from 11.7 μg/g dw β-CE in the first generation to 75.1 μg/g dw β-CE in the fourth generation. Qualitative and quantitative phenotypic and agronomical data have been recorded for all the lines tested and has revealed some interesting trends (Paul et al., 2017). The origin of the transgene had the biggest impact with, for example, banana lines expressing the banana *MtPsy2a* appearing normal with no variation in critical agronomical features such as yield and cycle time. In contrast, although expression of the maize *Psy1B73* gene resulted in increased fruit PVA levels, many lines with undesirable phenotypes such as stunting and photo bleaching were observed.

**Table 2:** List of new promoters and genes tested in AFT-2 of the Banana21 project.

Promoter abbreviation	Full name	GenBank	Source	Specificity
Exp4	Expansin 4	Not available	*Musa acuminata* cv. Cavendish	Constitutive
Ext	Extensin	Not available	*Musa acuminata* cv. Cavendish	Constitutive
MT2A	Metallothionein	Not available	*Musa acuminata* cv. Cavendish	Constitutive

**Gene abbreviation**	**Protein**	**GenBank**	**Source**	**Function**

*DXS*	Deoxy-xylulose-5-phosphate synthase	NM_117647.3	*Arabidopsis thaliana*	Carotenoid biosynthesis
*ZmPsy1Q60*	Phytoene synthase	U32636	*Zea mays* cv. Q60	Carotenoid biosynthesis
*LYCB*	Lycopene β-cyclase	XM_015771749	*Oryza sativa*	Carotenoid biosynthesis


During Phase 2, scientists at NARO had established regenerable ECSs, as well as efficient transformation protocols, for two varieties, “M9” and “Sukali Ndiizi.” These breakthroughs allowed the generation of transgenic banana plants from both varieties containing either the *ZmPsy1B73* or the “Asupina”-derived phytoene synthase 2a (*MtPsy2a*) transgenes. Since the results of AFT-1 were not available when this activity commenced, these gene were placed under the control of the banana-derived Exp1 promoter (Mbabazi, 2015). The transgenic lines generated from both cultivars were subsequently grown in a field trial at NARL-NARO, Kawanda Uganda in 2010 to assess the fruit PVAC levels. This first Ugandan field trial (UFT-1), conducted under authorization from the Ugandan National Biosafety Committee (NBC), was a very important milestone for Banana21 as it represented the first GM crop field trial in sub-Saharan Africa where the events had been created by local scientists from a national laboratory.

At the completion of Phase 2, proof-of-concept for PVA biofortification had been demonstrated in Australia using “Cavendish” and “Lady Finger” bananas and revealed that target fruit PVA levels could be achieved using the constitutive (Ubi) or fruit-specific (Aco) expression of *MtPsy2a* alone (Paul et al., 2017). Further, very few off-type traits, such as reduced yield and increased cycle time, were observed in the transgenic lines and the trait appeared stable over successive generations. Importantly, the field trial in Uganda (UFT-1) produced multiple lines with fruit PVA levels higher than their respective controls including one Exp1-*MtPsy2a* line of M9 with 33.1 μg/g dw β-CE (Mbabazi, 2015). With this knowledge, a new generation of plant expression vectors were made at QUT and two constructs containing Ubi-*MtPsy2a* and Aco-*MtPsy2a* were transferred to NARO for transformation into EAHB varieties.

### Phase 3 – Product Development: Early Events Selection Field Trial in Uganda

This phase of the project (2012–2017) initially involved generating a total of 200 independent transgenic lines each of EAHB cultivars “M9” and “Nakitembe” containing *MtPsy2a* under the control of either the Ubi or the Aco promoters. Following molecular characterization by the now fully trained technical staff and highly qualified scientists at NARO, the transgenic lines were field planted (UFT-2) at NARL-NARO, Kawanda in August 2014 to identify suitable elite lines for further testing in multi-location field trials (MLTs). During those 5 years, the NARO team showed exceptional professionalism in learning and implementing good practices around generating, handling, and tracking GM products. The results from UFT-2, which will soon be published, clearly demonstrate that fruit PVACs can consistently accumulate at levels above the required target of 20 μg/g dw β-CE in GM-biofortified “M9” and “Nakitembe” without phenotypic alteration of the plants (**Figure 2B**).

From the current transgenic line selection trial at Kawanda, 10 elite lines each of “M9” and “Nakitembe” will be selected to progress through to future MLTs. The initial selection process began in 2017 by selecting lines with fruit PVA levels equal or greater than 20 μg/g dw β-CE at the full green developmental stage (harvesting stage in Uganda) and yield within 20% of non-GM controls plants. From this initial selection, molecular analysis was used to identify lines containing fewer than three copies of the integrated expression cassettes with a preference for single integrations. Although selection of “single copy” events is preferred in seed crops to produce homozygous lines that do not segregate for the transgenic trait in future generations, in a vegetatively propagated crop such as banana, it is only rationalized to increase the likelihood of events with “clean insert.”

During this phase, NARO scientists also conducted a preliminary blind sensory panel test of traditionally prepared banana meal (matooke) using fruit from both non-GM and PVA-biofortified “M9” and “Nakitembe” to compare appearance and texture but not taste. Interestingly, 80% of the panelists (*n* = 15) rated fruit from one of the “M9” PVA-biofortified lines as the most preferred whereas fruit from its non-GM counterpart had the highest dislike proportion (29%).

On the basis of these encouraging results, Banana21 entered its fourth and final phase of funding in October 2017. This final phase is focused on generating all the data required for the deregulation of a GM PVA-biofortified EAHB in Uganda. The prospect of the release of the world’s first deregulated GM banana developed from an African laboratory is as much exciting as it is daunting.

## The Future Challenges of Banana21 – the Not So Long Road Ahead to Deregulation

From a technology perspective, the groundwork in Uganda has been completed and the exceptional results from UFT-2 have provided the ideal platform to further select elite lines to progress through to MLTs and ultimately to farmer release.

Prior to MLTs, critical data necessary for the compilation of a deregulation dossier must be obtained. Whole-genome sequencing of the pre-selected lines is necessary to allow the site(s) of transgene insertion into the host genome to be identified. This is necessary to ensure that (i) the transgene is intact, (ii) there are no new open-reading frames created, (iii) there is no disruption of endogenous open-reading frames, and (iv) no plasmid sequence has been integrated into the host genome. Finally, it is essential that the composition of the fruit from the deregulated lines is very similar to that derived from the wild type. Therefore, compositional analysis for food characteristics such as calories, calories from fat, carbohydrates, protein, ash, and moisture will be done, and lines with values >15% different to controls will be discarded.

The next phase of the project involves the identification of two lines (one lead and one reserve) each of “M9” and “Nakitembe” from the MLTs, and obtaining the necessary agronomic, biochemical, and molecular data to ultimately prepare the dossier for submission to regulators in Uganda for deregulation and general release. At the end of this phase of the project (December 2021), these four lines are expected to meet all the agronomic, biochemical, and molecular analyses and biosafety assessment required for deregulation in Uganda under the proposed Biosafety Bill.

One of the major hurdles for Banana21 is the lack of a regulatory framework for biotechnology in Uganda. Without a regulatory body controlling the safe application of biotechnologies, Banana21 will not be able to release PVA-biofortified “Golden bananas” to farmers and consumers in need. After ratifying the Cartagena Protocol on Biosafety in 2002, it took 6 years for Uganda to approve a policy on Biotechnology and Biosafety in 2008. Under the current policy, the NBC supervises all GMO activities up to the stage of Confined Field Trial (CFT) under the supervision of the National Council of Science and Technology (UNCST) Act 1990. Therefore in 2012, the National Biotechnology and Biosafety Bill, 2012, was introduced into parliament to provide a regulatory framework and guide the implementation of modern biotechnology in Uganda to minimize any potential risks to the environment, human, and animal health. After 5 years, Uganda’s Parliament passed the National Biotechnology and Biosafety Bill into law becoming the Biosafety Act, 2017 on October 4, 2017. The new law will not only benefit Banana21 but also a multitude of other biotechnology products developed in Uganda such as bananas with bacterial wilt resistance, drought tolerant maize, bollworm resistance and herbicide tolerant cotton, and new cassava varieties with resistance to Cassava mosaic and brown streak viruses.

Our initial target of 20 μg/g dw β-CE was calculated using a bioconversion factor of PVACs to retinol of 6:1 based on the results of a study using Mongolian gerbils (Bresnahan et al., 2012). Underestimating this bioconversion factor would raise the target above its current value. For this reason, Banana21 with financial help form HarvestPlus and the BMGF, commissioned a nutrition study at Iowa State University to determine a more accurate bioconversion ratio in humans. Although the results from this study are not yet available, some of the PVA-biofortified lines that have been developed under Banana21 have over four-times the initial target value with no yield penalty and could potentially be substituted if all other deregulation criteria are met.

The development of robust diagnostics for banana viruses was another important component of this project. These diagnostics form the basis of a banana virus indexing protocol that has been rolled out to ensure that the plantlets derived from the Banana21 project are virus tested, thus reducing the potential distribution of infected planting material to farmers.

Since the majority of banana growers in Uganda are subsistence farmers, the distribution strategy adopted in the future will need to minimize the cost of planting material while maximizing the rate of distribution. Initial propagation of the lines to be released will be done at NARO and the plantlets will be sent to small banana micropropagation laboratories and also used to establish small “mother gardens” for the initial production of suckers. A cost-effective and self-sustaining strategy for dissemination will then involve identifying “innovative farmers” that will be given suckers. For every sucker, they will be asked to give away two suckers to neighbors who in turn will be asked to give away two suckers for each one received under the scheme.

A key component of the next phase of the project will be the implementation of a comprehensive stewardship and communication plan. This includes (i) forming a Technical Advisory Committee (TAC) that meets regularly and provides scientific, strategic, and biosafety expertise, (ii) implementing and regularly updating SOPs, (iii) keeping accurate and safe records of the data with tools such as the BananaTracker software developed by QUT, and (iv) meetings with the Australian OGTR and Food Standards Australia and New Zealand (FSANZ) to seek advice on the requirements for deregulation if these lines were to be deregulated in Australia. The NARO team has also been involved in various communication activities in an attempt to educate the public and de-mystify the use of GMOs. Important stakeholders are targeted through workshops and information sessions, as well as various paper-based and audio-visual communication materials.

## Final Remarks and Conclusion

From the outset in 2005, Banana21 has been on a trajectory to develop lines of EAHBs with levels of fruit PVACs that would provide 50% of the EAR of VA with consumption of only 300 g per person per day. Based on the significant progress thus far, it is highly likely that the transgenic lines developed under Banana21 will be released by 2021 and have a significant impact in alleviating VAD in a sustainable way, especially in rural Uganda where bananas are a fundamental part of the culture. The PVA-enhanced, disease-resistant “M9” line will have the greatest impact in lower elevations of Uganda where the disease pressure is high, while the PVA-enhanced “Nakitembe” line will have greatest impact in the highlands where there is much lower disease pressure. The importance of banana as a food security crop (perennial nature, year-round production, and ability to cope with long periods of drought) associated with a low cost, farmer-driven distribution strategy should ultimately see “Golden bananas” adopted as a widespread and efficient VAD alleviating strategy in the next decade.

## Author Contributions

J-YP and RH drafted the initial manuscript while WT and JD reviewed and provided the constructive criticisms.

## Conflict of Interest Statement

The authors declare that the research was conducted in the absence of any commercial or financial relationships that could be construed as a potential conflict of interest.

## Footnotes

1 http://www.banana21.org/

2 http://www.ogtr.gov.au/internet/ogtr/publishing.nsf/Content/dir076rarmp-toc~dir076rarmp-ch1~dir076rarmp-ch1s5
